# Orbitofrontal cortex mediates the differential impact of signaled-reward probability on discrimination accuracy

**DOI:** 10.3389/fnins.2015.00230

**Published:** 2015-06-23

**Authors:** Ryan D. Ward, Vanessa Winiger, Eric R. Kandel, Peter D Balsam, Eleanor H. Simpson

**Affiliations:** ^1^Department of Neuroscience, Columbia UniversityNew York, NY, USA; ^2^Howard Hughes Medical InstituteChevy Chase, MD, USA; ^3^Kavli Institute for Brain Science, Columbia UniversityNew York, NY, USA; ^4^Department of Psychiatry, Columbia UniversityNew York, NY, USA; ^5^Department of Psychology, Barnard CollegeNew York, NY, USA; ^6^New York State Psychiatric InstituteNew York, NY, USA

**Keywords:** sustained attention, motivation, cognition-motivation interactions, orbitofrontal cortex, DREADD, pharmacogenetic inhibition, signaled-reward probability, discrimination accuracy

## Abstract

Orbitofrontal cortex (OFC) function is critical to decision making and behavior based on the value of expected outcomes. While some of the roles the OFC plays in value computations and behavior have been identified, the role of the OFC in modulating cognitive resources based on reward expectancy has not been explored. Here we assessed the involvement of OFC in the interaction between motivation and attention. We tested mice in a sustained-attention task in which explicitly signaling the probability of reward differentially modulates discrimination accuracy. Using pharmacogenetic methods, we generated mice in which neuronal activity in the OFC could be transiently and reversibly inhibited during performance of our signaled-probability task. We found that inhibiting OFC neuronal activity abolished the ability of reward-associated cues to differentially impact accuracy of sustained-attention performance. This failure to modulate attention occurred despite evidence that mice still processed the differential value of the reward-associated cues. These data indicate that OFC function is critical for the ability of a reward-related signal to impact other cognitive and decision-making processes and begin to delineate the neural circuitry involved in the interaction between motivation and attention.

## Introduction

It is well known that knowledge of the value of a potentially-earned reward can impact performance in cognitive tasks. Explicitly signaling changes in reward value influences discrimination accuracy in monkeys (Leon and Shadlen, 1999; Bendiksby and Platt, 2006), pigeons (Jones et al., 1995; Brown and White, 2005), and humans (Engelmann and Pessoa, 2007; Engelmann et al., 2009). Yet, little is known about the circuits underlying how representations of expected reward impact cognitive performance.

The orbitofrontal cortex (OFC) is involved in the representation of reward as demonstrated in its critical role in value-based decision making (Schoenbaum et al., 2009). Recent research suggests that OFC does not encode value *per se*, but is involved in adaptive decision making that requires information about the value of specific outcomes, particularly when this information must be dynamically updated and used to guide selection of specific behaviors that lead to those outcomes (Schoenbaum et al., 2011; Takahashi et al., 2013).

The aim of the present research was to see if OFC modulates the recruitment of cognitive resources based on reward expectations. Specifically, we asked if the OFC plays a role in the modulation of discrimination accuracy when explicit cues signal changes in the likelihood of reward. Altered performance under these conditions is thought to reflect differences in the top-down recruitment of attention to trial-specific stimuli (Corbetta and Shulman, 2002; Pessoa et al., 2003; Small et al., 2005; Pessoa and Engelmann, 2010).

To investigate the role of the OFC in modulation of discrimination accuracy in response to reward-associated cues, we generated mice in which neuronal activity in the OFC could be transiently (for the duration of a single behavioral test session) silenced. This was achieved by stereotaxically injecting a virus which drives expression of the Designer Receptor Exclusively Activated by Designer Drug (DREADD) hM4D(G_i_) selectively in neurons. Systemic administration of the synthetic drug clozapine-N-oxide (CNO) induces G_i_ activation which mediates decreased neuronal activity selectively in neurons in which the hM4D(G_i_) receptor is expressed (Armbruster et al., 2007).

These mice were tested in a procedure which explicitly assays the impact of motivation on attention (Ward et al., 2015). In our signaled-probability sustained-attention task (modeled after the five-choice serial reaction-time task; Robbins, 2002), the correct response on a given trial is a lever press which is cued by a stimulus light. As with the 5CSRTT, we have previously shown that increasing attentional demand by decreasing the duration of cue presentation worsens discrimination performance (Kahn et al., 2012; Ward et al., 2015). Motivation to attend during the task is manipulated by explicitly signaling the probability of reward for correct choice responses on a trial-by-trial basis. Under control conditions, mice performed with greater accuracy when the signaled-reward probability was high. When OFC neuronal activity was inhibited, discrimination performance was not modulated by cues associated with different reward probabilities. The inhibition did not eliminate the representation of differential-outcome likelihood associated with different cues but specifically interfered with the capacity for this information to influence attention or decision processes.

## Methods and materials

### Mice

Mice were male F1 hybrids (3–6 months old at the beginning of the experiment) of the C57BL/6J and 129Svev (Tac) background strain. Mice were housed, bred, and tested in compliance with the New York State Psychiatric Institute and Columbia University Institutional Animal Care and Use Committees.

### Apparatus

Operant chambers (Med-Associates, St. Albans, VT; model ENV-307w) were used in all behavioral testing. The operant chambers had internal dimensions 22½ × 18½ × 12½ and were located in a light- and sound- attenuating cabinet equipped with an exhaust fan, which provided 72 dB background white noise. Each chamber was equipped with a feeder trough that was centered on one wall of the chamber. A reward of one drop of evaporated milk could be provided by raising a dipper. An infrared photocell detector was used to record head entries into the trough. Two retractable levers were mounted on the same wall as the feeder trough. The chambers were illuminated throughout all sessions with a houselight (Med Associates #1820) located at the top of the chamber. An audio speaker was positioned 8.5 cm from the floor on the wall opposite the feeder trough. The speaker delivered a brief tone (90 db, 2500 Hz, 200 ms) to signal when the liquid dipper was raised.

### Experimental procedures

#### Sustained-attention task

All training and testing sessions occurred once per day, 7 d per week. Animals were first trained to consume evaporated milk from the liquid dipper. The mice were then trained to press the lever to obtain rewards on a continuous reinforcement (CRF) schedule as described previously (Ward et al., 2012). Each CRF session ended after 60 rewards or 60 min, whichever occurred first. Subjects that had earned fewer than 30 rewards on the third day of CRF training were given an overnight (14-h) session with no limit on earned rewards. Discrimination training then occurred in several phases. In all phases, each trial began with an intertrial interval (ITI) of unpredictable duration (mean = 45 s, range 2.74–148.13 s).

#### Single cue-single lever training

During single cue-single lever training, mice received trials where a cue light above a lever on either the left or right side of the chamber was illuminated for 10 s. One second after the cue's termination, the lever beneath the cued light was presented for 10 s. Pressing the lever beneath the cued light resulted in a dipper reward. The cue light/lever position alternated daily across a total of four sessions, until the mice reliably pressed the lever after each stimulus cue presentation.

#### Choice training

During choice training, a percentage of the trials were single cue-single lever trials as described above, while the remaining percentage were choice trials. The position of the cue light (left or right) was randomly determined from trial to trial. During choice trials, both of the levers were inserted 1 s after the cue's termination, and a response to the lever that had been cued at the beginning of the trial was rewarded. Incorrect responses resulted in a correction procedure, where the trial was repeated with the cue light in the same location until a correct response was made. Training consisted of three sessions with 50% choice: 50% single lever-cue trials, three sessions of 80% choice: 20% single lever-cue trials, and nine sessions of 100% choice trials, all with correction. This was followed by 10 sessions of 100% choice trials without correction. During these sessions, incorrect responses resulted in both levers being withdrawn and a new trial being initiated. If no response was made after 10 s (an omission), both levers were retracted and a new trial began.

We have shown previously that accuracy on this task is sensitive to increasing attentional demand (i.e., accuracy decreases with decreasing cue duration; Kahn et al., 2012; Ward et al., 2015). Thus, it is a sensitive assay with which to test manipulations that impact attention.

#### Signaled-probability sustained-attention task

Following acquisition of the sustained-attention task, mice were moved to the signaled-reward probability sustained-attention task, in which the probability of reward for a correct choice response (either 1.0 or 0.1) on each upcoming trial was signaled by either turning the houselight on or off during the trial (counterbalanced across mice). Mice received an equal number of high and low reward-probability trials. High and low reward-probability trials were presented pseudorandomly with the constraint that no more than four consecutive trial types of the same reward probability could be presented in a row. Mice received six sessions on this task, after which the cue duration was successively decreased from 10 to 2 s over the course of 15 sessions.

#### Viruses and stereotaxic injection protocol

Viruses were obtained from the University of North Carolina Gene Therapy Center Vector Core. Mice were stereotactically injected bilaterally with either AAV2/hSyn-HA-hM4D(G_i_)-IRES-mCitrine [3 × 10^12^ particles/ml; hereafter referred to as hM4D(G_i_)] or AAV2/hSyn-eGFP (4 × 10^12^ particles/ml; hereafter referred to as GFP). Viruses (0.5 μL) were pressure injected using a glass pipette (9–12 μm) into the OFC (coordinates: +2.60 mm anterior to bregma; ±1.10 mm medial and lateral to midline; 1.80 mm below brain surface).

hM4D(G_i_) is a modified human muscarinic receptor that does not bind endogenous ligands but responds to a synthetic compound, clozapine-n-oxide (CNO). When CNO binds to hM4D(G_i_), it produces a hyperpolarization of the cell through a g-protein mediated activation of inward-rectifying potassium channels (Armbruster et al., 2007). We have successfully used this method previously to silence neurons *in vivo* in behaving mice (Parnaudeau et al., 2013, 2015). Importantly, this method has also been shown recently to significantly inhibit activity of OFC neurons in behaving mice (Gremel and Costa, 2013).

Mice with bilateral orbitofrontal cortex GFP (*N* = 12) or hM4D(G_i_)-mCitrine (*N* = 11) viral injections were tested in the signaled-reward probability sustained-attention task. After cue duration was decreased to 2 s, mice received several i.p. injections to accustom them to the injection procedure. Following this, mice received injections of saline and CNO, counterbalanced for order, in a within-subjects design, in which all mice received both types of injections.

#### Drugs and injection protocol

CNO (obtained from NIH) was dissolved in saline to a final concentration of 0.2 mg/ml. Saline or CNO (2 mg/kg) was administered intraperitoneal to the mice 30 min before behavioral testing. This dose was chosen based on our previous work using the DREADD method and has been shown to significantly reduce neuronal firing in infected neurons *in vitro* and this impacted both cognition and also task related synchronous activity with a distal structure during *in vivo* recordings (Parnaudeau et al., 2013, 2015). After an additional three sessions of drug-free testing, this injection regimen was repeated so that there were two determinations of the drug effect in each subject. For three mice in each group, an equipment malfunction resulted in data not being correctly recorded during one of the saline or CNO sessions during the first injection regimen. In these cases, the obtained value reflects only the data from the second injection regimen. For the mice on whom we had data from both drug determinations, we conducted a reward probability (high vs. low) × treatment (saline vs. CNO) × viral injection [GFP vs. hM4D(G_i_)] × drug determination ANOVA on the accuracy data which showed that the overall effect of determination was not significant [effect of drug determination; *F*_(1, 18)_ = 0.132, *p* = 0.720], nor were any of the interactions between determination and the other factors (*Fs* < 0.70). Because there was no statistically significant difference in the data obtained from the two determinations, data reported below represent the average of the two drug-testing regimens.

### Data analysis

The main dependent measure of interest was the proportion of correct responses. We also analyzed latency to make a choice response, latency to retrieve rewards, proportion of trials omitted, and the proportion of total responses made on the previously correct lever, as well as the number of errors made on the previously correct lever (measures of perseverative responding). For statistical comparison, repeated-measures analyses of variance with appropriate factors, followed by Bonferroni post-tests were used. Individual means were compared using paired-samples *t*-tests. Latency to retrieve rewards was compared using a between-subjects *t*-test.

## Results

### Pharmacogenetic inhibition of OFC function abolishes the impact of reward-associated cues on attention

**Figure 1:**
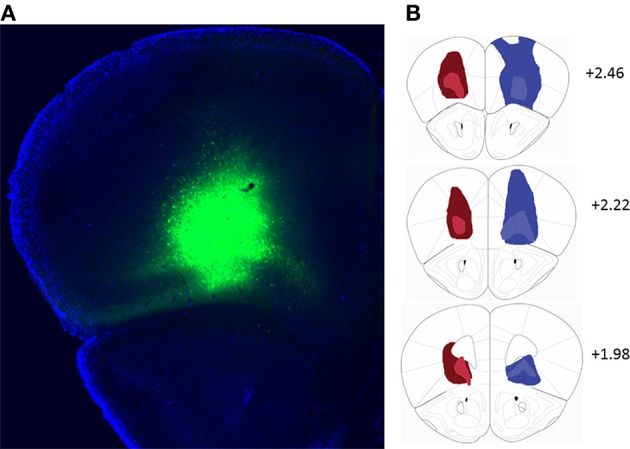
**(A)** Representative example of viral expression in the orbitofrontal cortex. **(B)** Diagrammatic representation of the spread of hM4D(G_i_)-mCitrine (red) and GFP (blue) virus. All injections were bilateral. For clarity, the minimal (light colors) and maximal (dark colors) extent of each type of injection is depicted only on one hemisphere. Numbers indicate relative distance from bregma according to Paxinos and Franklin (2001). hM4D(G_*i*_) *N* = 11, GFP *N* = 12.

Bilateral stereotaxic injection of either hM4D(G_i_)-mCitrine or GFP expressing adeno-associated viruses resulted in expression of either hM4D(G_i_) and mCitrine or GFP selectively in neurons due to the use of the human Synapsin1 promoter (hSyn). Figure 1A shows a representative image of viral expression in OFC. The minimal and maximal extent of intrinsic fluorescence of mCitrine [from the hM4D(G_i_) expressing virus] and GFP in either hemisphere are depicted in the left and right hemispheres, respectively, of coronal sections in Figure 1B. Intrinsic fluorescence was largely located in lateral and ventral orbitofrontal cortices. In a few cases in GFP-injected control mice there was some spreading of the virus to M1, M2, and frontal association cortex.

**Figure 2:**
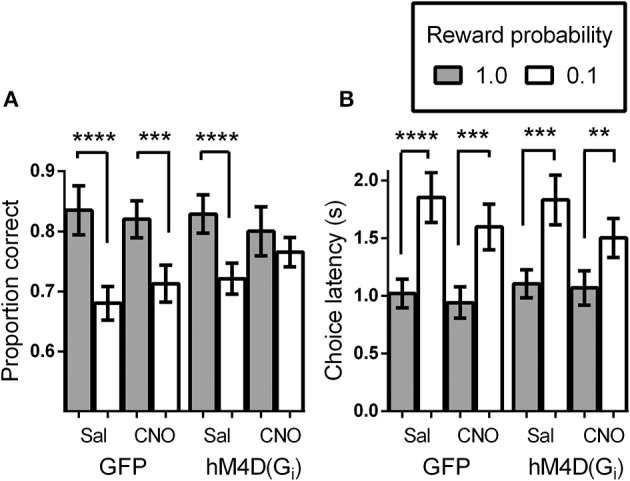
**(A)** Proportion correct as a function of signaled-reward probability for GFP and hM4D(G_i_) mice treated with saline and CNO. **(B)** Choice response latencies as a function of signaled-reward probability for GFP and hM4D(G_i_) mice treated with saline and CNO. hM4D(G_i_) *N* = 11, GFP *N* = 12. **p < 0.01, ***p < 0.001, ****p < 0.0001.

We tested virally injected mice in the signaled-reward probability sustained-attention task after injection of either vehicle (saline), or CNO. A reward probability × viral injection × treatment ANOVA on proportion correct indicated that the effects of viral injection [GFP vs. hM4D(G_i_)] and treatment (saline vs. CNO) were not significant [*F*_(1, 21)_ = 0.295, *p* = 0.593 and *F*_(1, 21)_ = 0.091, *p* = 0.766, respectively]. There was a significant effect of reward probability [*F*_(1, 21)_ = 34.85, *p* = 0.000], and significant reward probability × treatment [*F*_(1, 21)_ = 8.52, *p* = 0.008] interaction. No other interactions were significant. To specify the nature of the significant interactions, we conducted separate ANOVAs on the data from the GFP and hM4D(G_i_) groups. Figure 2A shows that, in GFP mice, signaling the probability of reward had a significant impact on discrimination accuracy [effect of signaled probability; *F*_(1, 11)_ = 25.79, *p* = 0.00] which was not differentially affected by saline or CNO treatment [effect of treatment; *F*_(1, 11)_ = 0.057, *p* = 0.82; probability × treatment interaction; *F*_(1, 11)_ = 2.71, *p* = 0.13]. By contrast, in hM4D(G_i_) mice, there was also a significant impact of signaled-reward probability on discrimination accuracy [*F*_(1, 10)_ = 10.41, *p* = 0.009], but silencing OFC activity via CNO treatment eliminated the effect of signaled-reward probability on discrimination accuracy (probability × treatment interaction); [*F*_(1, 10)_ = 6.16, *p* = 0.03] without impacting overall discrimination accuracy [effect of treatment; *F*_(1, 10)_ = 0.036, *p* = 0.85]. Planned *post-hoc* comparisons showed that accuracy during high-reward probability and low-reward probability trials was significantly different for hM4D(G_i_) mice treated with saline [*t*_(10)_ = 6.15, *p* = 0.000], but not with CNO [*t*_(10)_ = 1.05, *p* = 0.32].

### Intact encoding of signaled-reward probability in mice during OFC inhibition

In addition to analyzing the effect of signaled-reward probability on response choice, we analyzed the latency to make a choice response during the task (Figure 2B). Trials on which mice failed to respond were not included in these calculations. As above, the overall effect of viral injection [*F*_(1, 21)_ = 0.021, *p* = 0.89] and treatment [*F*_(1, 21)_ = 1.28, *p* = 0.27] were not significant, but there was a significant effect of reward probability [*F*_(1, 21)_ = 70.48, *p* = 0.000] and a significant reward probability × treatment interaction [*F*_(1, 21)_ = 5.004, *p* = 0.036]. To further analyze performance, separate ANOVAs were conducted on the latency data from GFP and hM4D(Gi) mice. As shown in Figure 2B, latency to respond was shorter on high-reward probability trials than on low-reward probability trials [effect of reward probability; *F*_(1, 11)_ = 43.45, *p* = 0.00 and *F*_(1, 10)_ = 28.12, *p* = 0.000] for GFP and hM4D(G_i_) mice, respectively. Differently from the effect on discrimination accuracy, however, there was no significant effect of OFC neuronal silencing on the latency to make a choice response. Although latencies became noticeably shorter on low reward probability trials under CNO treatment, this effect was not statistically significant in either GFP or hM4D(G_i_) mice [reward probability × treatment interaction *F*_(1, 11)_ = 3.64, *p* = 0.09 and *F*_(1, 10)_ = 1.51, *p* = 0.25, respectively]. These results demonstrate that OFC inactivation did not eliminate the ability to associate different reward probabilities with specific cues nor did it impair an overall ability to use that information in motivated behavior. We also analyzed latency to retrieve rewards and found no difference between GFP or hM4D(G_i_) mice treated with either saline or CNO (*ps* > 0.50).

### OFC inhibition does not produce perseverative responding

**Figure 3:**
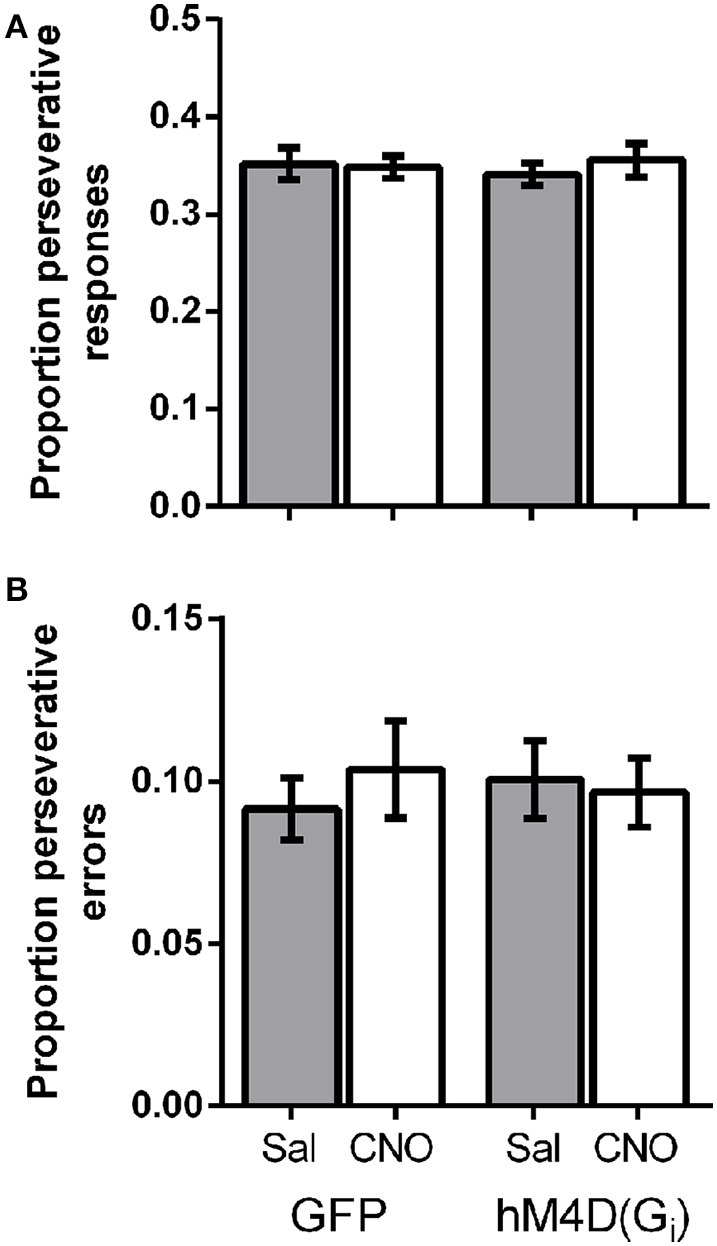
**(A)** Proportion of perseverative responses for GFP and hM4D(G_i_) mice treated with saline and CNO. **(B)** Proportion of perseverative errors for GFP and hM4D(G_i_) mice treated with saline and CNO.

Lesions of the OFC have been shown to impair reversal learning performance by producing perseveration on a previously rewarded response (Clarke et al., 2008). To determine whether OFC inactivation altered perseveration in the present study, we calculated the proportion of total responses that were made on the lever that was correct on the previous trial. Figure 3A shows that there was no difference in the proportion of perseverative responses between GFP and hM4D(Gi) injected mice [effect of viral injection; *F*_(1, 21)_ = 0.015, *p* = 0.904]. There was also no impact of treatment (saline vs. CNO) on perseverative responses [*F*_(1, 21)_ = 0.18, *p* = 0.678], and no interaction between viral injection and treatment [*F*_(1, 21)_ = 0.451, *p* = 0.509]. We also calculated the proportion of perseverative errors (incorrect responses made to the previously correct lever; Figure 3B). Again, there was no difference in the proportion of perseverative errors between GFP and hM4D(Gi) injected mice [effect of viral injection; *F*_(1, 21)_ = 0.010, *p* = 0.923]. Similarly, there was also no impact of treatment (saline vs. CNO) on perseverative errors [*F*_(1, 21)_ = 0.10, *p* = 0.755], and no interaction between viral injection and treatment [*F*_(1, 21)_ = 0.380, *p* = 0.544]. These results indicate that OFC inactivation did not produce impairments by increasing perseverative responding.

### OFC inhibition does not impact motivation to participate in the task

**Figure 4:**
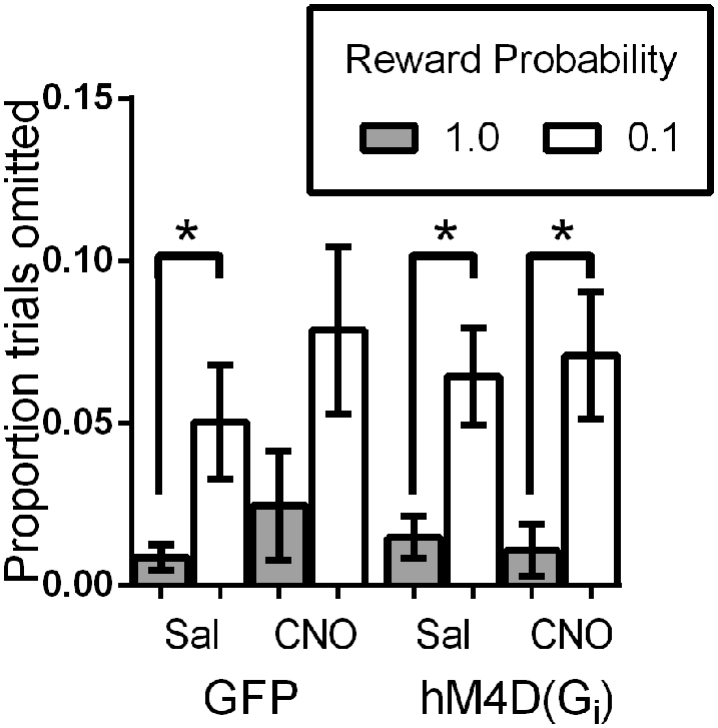
Proportion of trials omitted as a function of signaled-reward probability for GFP and hM4D(G_i_) mice treated with saline and CNO. * ***p*** **< 0.05**.

We also analyzed the proportion of trials on which mice did not make a choice response to determine whether OFC inactivation impacted motivation to engage in the task. Figure 4 shows that overall, mice completed the majority of trials (>90%). As we have previously reported (Ward et al., 2015), mice omitted responses on significantly more low reward-probability trials than high probability trials, indicating decreased motivation to engage in these trials [effect of reward probability; *F*_(1, 21)_ = 13.99, *p* = 0.001]. There was no effect of viral injection [GFP vs. hM4D(Gi); *F*_(1, 21)_ = 0.001, *p* = 0.981] or treatment [saline vs. CNO; *F*_(1, 21)_ = 2.73, *p* = 0.114], and none of the interactions were significant. These results indicate that mice were less motivated to respond on low reward-probability trials, but OFC inhibition did not impact the proportion of omitted trials.

## Discussion

Transient inhibition of neuronal activity in the OFC attenuated the ability of reward-related cues to modulate differential discrimination accuracy. Importantly, neither the presence of the hM4D(Gi) receptor or CNO alone had any impact on accuracy. It was only when the hM4D(Gi) receptor was activated by CNO that the effects were seen. This effect was not the result of an increase in perseverative responding. Furthermore, because overall accuracy was not impaired, this occurred in the absence of general decrements in attention. Additionally, the mice appreciated that different cues signaled different reward probabilities, as evidenced by the fact that both choice-response latencies and overall task engagement were modulated by the signals. Thus, inhibiting the OFC did not impact (1) overall attention; or (2) encoding of the relation between signals and outcome probability *per se*, but impaired the ability of the mice to use that information to modulate attention or other processes that impact discrimination accuracy.

Recent work parsing the role of the OFC in behavior and decision making has indicated that the OFC may not be necessary in simple value-based behavior, but that it is critical when information about specific outcomes is relevant to ongoing choice behavior and decision making (Schoenbaum et al., 2009; Walton et al., 2010; Takahashi et al., 2013). The signaled-reward probability paradigm employed here involves learning the visual discrimination, learning that the different cues signal different reward probabilities, and then leveraging that knowledge to differentially recruit attentional processes. The dissociation between the lack of effect of signaled-reward probability on discrimination accuracy in mice during OFC inhibition, and the spared differential effect of reward probability on choice-response latencies and omitted trials, is further support for the distinction of the specific psychological processes subserved by OFC. Specifically, our data suggest that OFC is not required for a probability signal to modulate differential behavioral responses *per se*; rather the OFC is critically involved in the ability of that same probability signal to modulate other cognitive and decision-making processes.

Although we show here that the OFC might be critical for the ability of reward-associated cues to differentially impact attention, the present data do not demonstrate that OFC is sufficient for such modulation. A growing body of work has deepened understanding of the subtle and complex role of the OFC in this type of decision making (Furuyashiki et al., 2008), and has elucidated the critical role of connectivity with other structures in value-based behavior. For example, interactions between OFC and basolateral amygdala have been shown to be critical for using information based on the learned value of outcomes (Schoenbaum et al., 1998, 1999, 2007; Baxter et al., 2000; Blundell et al., 2001; Saddoris et al., 2005).

We have thus far interpreted the results from our signaled-probability task as being indicative of differential attentional recruitment in response to reward-associated cues. Our previous data (Ward et al., 2015) using this task suggest that differential accuracy on high and low-probability trials is most pronounced at shorter cue durations, suggesting that the ability of the reward-probability signal to recruit attention depends on how taxed attentional resources are by current task requirements. The current results suggest that the OFC may be necessary under these conditions to modulate differential recruitment of attention in response to reward-associated cues. This interpretation may be consistent with recent results which show that the OFC may play a role in attention in addition to its role in value-based decision making (Chase et al., 2012), and that the OFC signals the increased salience of situations in which multiple outcomes (in our case, high and low reward probability) are expected (Ogawa et al., 2013). We should note, however, that while our task requires the interaction of motivation and attention on some level, we cannot unequivocally conclude that differential accuracy on high and low reward-probability trials can only be understood in terms of top-down recruitment of attention, or that OFC inhibition compromises this specific aspect of performance. Such confirmation would require parametric manipulation of cue duration and reward probability combined with OFC inactivation.

Another interpretation of the obtained results is that given the increased latency to respond on low-probability trials, these trials taxed working memory more than high-probability trials, and the differences in accuracy are indicative of deficits in remembering the location of the cue. This interpretation is less plausible, however, given the fact that accuracy of mice in the present sustained-attention task is not impaired when explicit delays within the range of the latency intervals obtained here are inserted between cue presentation and presentation of choice-response levers (Ward et al., 2015). Thus, the difference in accuracy is not likely to be due to working memory for correct cue location being unduly taxed on low-probability trials.

Another alternative to the attentional recruitment account of our data is that the transient inhibition of neuronal activity in the OFC resulted in an inability to recruit motivational processes. Based on electrophysiology and human neuroimaging evidence, there is overlap in areas involved in the processing of reward and those involved in recruiting attention to motivationally-relevant stimuli (Pessoa and Engelmann, 2010). Thus, the degree to which motivational processes are distinct, both psychologically and at the functional neural level, from attentional processes in experiments like the one reported here may be difficult to specify. Indeed, some have suggested that motivation may exert its effects on behavior by engaging the same functional circuitry used by the attention system (Pessoa and Engelmann, 2010). However, the fact that reward probability was manipulated on a trial-by-trial basis, as opposed to over blocks of trials or over sessions, suggests that the differential accuracy was not due to general, bottom-up, arousal-mediated motivational effects. Furthermore, OFC inhibition did not change the proportion of trials omitted, indicating that it did not impact overall motivation to engage in the task. The present results suggest therefore, that if OFC inhibition impacted accuracy through motivation it must be through a process that regulates action on a trial-by-trial basis. There is ample evidence that OFC neurons code for outcomes when a signal indicates a specific outcome (Tremblay and Schultz, 1999; Padoa-Schioppa and Assad, 2006; van Duuren et al., 2007). Thus, it is possible that OFC inactivation interferes with the use of information about differential encoding of reward probability from trial-to-trial.

Recent research on the nature of the interactions between the nucleus accumbens (NAc) and the OFC in value-based decision making may suggest different specific roles of the OFC in the performance seen here. The NAc is widely known to be critical for reward-motivated behavior in a variety of paradigms (Salamone et al., 2007). Given the functional connectivity of the NAc with the basal forebrain and the medial prefrontal cortex, both critical components of the attentional machinery needed for our task, the NAc is particularly well situated to mediate the recruitment of attention via reward-associated cues (Hasselmo and Sarter, 2011). It also receives direct projections from the OFC, and recent work has clarified the nature of the interactions between NAc and OFC in value-based decision making. For example, Stott and Redish (2014) recorded concurrently from NAc and OFC during a spatial delay-discounting task that involved a trade-off between reward delay and magnitude. Importantly, they were able to isolate neural activity that occurred during deliberation, before the choice occurred, from activity which occurred after the choice was made. They found that activity in NAc signaled aspects of the reward before the choice was made (see also van der Meer and Redish, 2009), whereas activity in both NAc and OFC maintained representations of reward during the execution of the chosen response. Based on these results, they suggested that NAc is more directly involved in the planning of action during behavioral choice, while both NAc and OFC process information related to the decision, execution, and receipt of an outcome once the decision is made. Inactivation of OFC in our experiment could alter all of the later steps following the decision point and contribute to the inability to use information about reward to guide response selection.

Thus, is seems likely that the OFC is integrating information about both the cued correct choice location gained from employment of attentional processes (possibly facilitated by the NAc) and the signaled-reward probability to facilitate the accurate use of the information for response selection and execution of the selected response. An impairment in the capacity to maintain a representation of the integrated information would lead to less differential responding on high and low reward-probability trials.

The dissociation reported here between the impact of signaled-reward probability on choice-response latencies and discrimination accuracy is consistent with this interpretation of OFC function. Impaired ability to use information about reward probability to modulate response selection and execution accuracy on a trial-by-trial basis in the present procedure could be based on accurate encoding of reward probability but an inability to use that information to guide and/or sustain response choice. Thus, perhaps mice encoded the differential value of the reward-associated cues and this information impacted their choice-response latencies (they were more motivated to respond on high-probability trials), but they were unable to use this information either during cue presentation to recruit/direct attention appropriately or during the choice phase to adaptively modulate choice behavior. Although similar to the above interpretation, in that the role of the OFC is to allow for association of a particular reward value with a particular response, this interpretation differs from that proposed by (Stott and Redish, 2014 see also Walton et al., 2010) in that rather than altering an updating process which impacts choice behavior on subsequent trials, we suggest that OFC inhibition impacted the decision process by altering choice behavior on the current trial. In sum, we suggest that OFC may play critical roles in both the dynamic recruitment of attention in response to signaled-reward probability and/or in the selection and execution of a choice response.

A number of results suggest that there are likely separate and dissociable roles of the lateral and medial OFC in reward-motivated behavior and decision making (Burton et al., 2014; Rudebeck and Murray, 2014). Specifically, lateral OFC is thought to be involved in evaluating differences in expected outcomes, while medial OFC is involved in guiding choices based on the expected value of these outcomes (Rudebeck and Murray, 2014). Our viral injections included both medial and lateral OFC, but were biased toward medial OFC. The dissociation between the impact of signaled-reward probability on choice-response latencies and discrimination accuracy may reflect inhibition of the choice-modulating function of the medial OFC, but spared valuation by lateral OFC. Further work is needed to delineate the specific roles of distinct anatomical areas of OFC in this task.

These results demonstrate that normal OFC function is necessary for the ability of motivationally-significant cues to impact cognitive performance. Deficits in motivation and cognition are present in diseases such as schizophrenia, and severity of these deficits determines functional outcomes and quality of life (Bowie and Harvey, 2006; Green, 2006). Additionally, cognition and motivation interact to produce dysfunction, at least in part through an inability to adaptively modify behavior in response to motivationally-significant cues (Barch, 2005; Nakagami et al., 2008). Numerous results point to prefrontal dysfunction in the pathophysiology of schizophrenia (Berman et al., 1988; Crespo-Facorro et al., 2000; Wang et al., 2014). Indeed, cognitive deficits described in patients are prototypical of the type of deficits seen when OFC function is compromised, including deficits in reversal learning (Waltz and Gold, 2007) and insensitivity of performance to variation in reward probability (Gold et al., 2012, 2013). Our results lend support to the hypothesis that OFC plays a causal role in the interaction of motivation and cognition and that a disruption of this function is a likely source of cognitive and functional impairment in patients.

## Author contributions

RW, PB, and ES conceptualized the research. RW and VW conducted the research. EK provided lab space, reagents, and other material support and consultation for the research. RW and VW analyzed data. RW, PB, and ES wrote the manuscript.

### Conflict of interest statement

The authors declare that the research was conducted in the absence of any commercial or financial relationships that could be construed as a potential conflict of interest.

## References

[B1] Armbruster B. N. Li X. Pausch M. H. Herlitze S. Roth B. L. (2007). Evolving the lock to fit the key to create a family of G protein-coupled receptors potently activated by an inert ligand. Proc. Natl. Acad. Sci. U.S.A. 104, 5163–5168. 10.1073/pnas.0700293104 17360345 PMC1829280

[B2] Barch D. M. (2005). The relationships among cognition, motivation, and emotion in schizophrenia: how much and how little we know. Schizophr. Bull. 31, 875–881. 10.1093/schbul/sbi040 16079388

[B3] Baxter M. G. Parker A. Lindner C. C. C. Izquierdo A. D. Murray E. A. (2000). Control of response selection by reinforcer value requires interaction of amygdala and orbitofrontal cortex. J. Neurosci. 20, 4311–4319. 10818166 10.1523/JNEUROSCI.20-11-04311.2000 PMC6772657

[B4] Bendiksby M. S. Platt M. L. (2006). Neural correlates of reward and attention in macaque area LIP. Neuropsychologia 44, 2411–2420. 10.1016/j.neuropsychologia.2006.04.011 16757005

[B5] Berman K. F. Illowsky B. P. Weinberger D. R. (1988). Physiological dysfunction of dorsolateral prefrontal cortex in schizophrenia. IV. Further evidence for regional and behavioral specificity. Arch. Gen. Psychiatry 45, 616–622. 10.1001/archpsyc.1988.01800310020002 3382321

[B6] Blundell P. Hall G. Killcross S. (2001). Lesions of the basolateral amygdala disrupt selective aspects of reinforcer representation in rats. J. Neurosci. 21, 9018–9026. 11698612 10.1523/JNEUROSCI.21-22-09018.2001 PMC6762271

[B7] Bowie C. R. Harvey P. D. (2006). Cognitive deficits and functional outcome in schizophrenia. Neuropsychiatr. Dis. Treat. 2, 531–536. 10.2147/nedt.2006.2.4.531 19412501 PMC2671937

[B8] Brown G. S. White K. G. (2005). On the effects of signaling reinforcer probability and magnitude in delayed matching to sample. J. Exp. Anal. Behav. 83, 119–128. 10.1901/jeab.2005.94-03 15828590 PMC1193742

[B9] Burton A. C. Kashtelyan V. Bryden D. W. Roesch M. R. (2014). Increased firing to cues that predict low-value reward in the medial orbitofrontal cortex. Cereb. Cortex 24, 3310–3321. 10.1093/cercor/bht189 23901075 PMC4224243

[B10] Chase E. A. Tait D. S. Brown V. J. (2012). Lesions of the orbital prefrontal cortex impair the formation of attentional set in rats. Eur. J. Neurosci. 36, 2368–2375. 10.1111/j.1460-9568.2012.08141.x 22672207

[B11] Clarke H. F. Robbins R. W. Roberts A. C. (2008). Lesions of the medial striatum in monkeys produce perseverative impairments during reversal learning similar to those produced by lesions of the orbitofrontal cortex. J. Neurosci. 28, 10972–10982. 10.1523/JNEUROSCI.1521-08.2008 18945905 PMC3981993

[B12] Corbetta M. Shulman G. L. (2002). Control of goal-directed and stimulus driven attention in the brain. Nat. Rev. Neurosci. 3, 201–215. 10.1038/nrn755 11994752

[B13] Crespo-Facorro B. Kim J. Andreason N. C. O'Leary D. S. Magnotta V. (2000). Regional frontal abnormalities in schizophrenia: a quantitative gray matter volume and cortical surface size study. Biol. Psychiatry 48, 110–119. 10.1016/S0006-2332(00)00238-9 10903407

[B14] Engelmann J. B. Damaraju E. Padmala S. Pessoa L. (2009). Combined effects of attention and motivation on visual task performance: transient and sustained motivational effects. Front. Hum. Neurosci. 3:4. 10.3389/neuro.09.004.2009 19434242 PMC2679199

[B15] Engelmann J. B. Pessoa L. (2007). Motivation sharpens exogenous spatial attention. Emotion 7, 668–674. 10.1037/1528-3542.7.3.668 17683222

[B15a] Furuyashiki T. Holland P. C. Gallagher M. (2008). Rat orbitofrontal cortex separately encodes response and outcome information during performance of goal-directed behavior. J. Neurosci. 28, 5127–5138. 10.1523/JNEUROSCI.0319-08.2008 18463266 PMC2693204

[B16] Gold J. M. Strauss G. P. Waltz J. A. Robinson B. M. Brown J. K. Frank M. J. (2013). Negative symptoms of schizophrenia are associated with abnormal effort-cost computations. Biol. Psychiatry 74, 130–136. 10.1016/j.biopsych.2012.12.022 23394903 PMC3703817

[B17] Gold J. M. Waltz J. A. Matveeva T. M. Kasanova Z. Strauss G. P. Herbener E. S.. (2012). Negative symptoms and the failure to represent the expected reward value of actions: behavioral and computational modeling evidence. Arch. Gen. Psychiatry 69, 129–138. 10.1001/archgenpsychiatry.2011.1269 22310503 PMC4406055

[B18] Green M. F. (2006). Cognitive impairment and functional outcome in schizophrenia and bipolar disorder. J. Clin. Psychiatry 67, 3–8. 10.4088/JCP.1006e12 16965182

[B19] Gremel C. M. Costa R. M. (2013). Orbitofrontal and striatal circuits dynamically encode the shift between goal-directed and habitual actions. Nat. Commun. 4:2264. 10.1038/ncomms3264 23921250 PMC4026062

[B20] Hasselmo M. E. Sarter M. (2011). Modes and models of forebrain cholinergic neuromodulation of cognition. Neuropsychopharmacology 36, 52–73. 10.1038/npp.2010.104 20668433 PMC2992803

[B21] Jones B. M. White K. G. Alsop B. L. (1995). On two effects of signaling the consequences for remembering. Anim. Learn. Behav. 23, 256–272. 10.3758/BF03198922

[B22] Kahn J. B. Ward R. D. Kahn L. Balsam P. D. Simpson E. H. (2012). Medial prefrontal lesions in mice impair sustained attention but spare maintenance of information in working memory. Learn. Mem. 19, 513–517. 10.1101/lm.026302.112 23073640 PMC3475157

[B23] Leon M. I. Shadlen M. N. (1999). Effect of expected reward magnitude on the response of neurons in the dorsolateral prefrontal cortex of the macaque. Neuron 24, 415–425. 10.1016/S0896-6273(00)80854-5 10571234

[B24] Nakagami E. Xie B. Hoe M. Brekke J. S. (2008). Intrinsic motivation, neurocognition, and psychosocial functioning in schizophrenia: testing mediator and moderator effects. Schizophr. Res. 105, 95–104. 10.1016/j.schres.2008.06.015 18715756

[B25] Ogawa M. van der Meer M. A. A. Esber G. R. Cerri D. H. Stalnaker T. A. Schoenbaum G. (2013). Risk-responsive orbitofrontal neurons track acquired salience. Neuron 77, 251–258. 10.1016/j.neuron.2012.11.006 23352162 PMC3559000

[B26] Padoa-Schioppa C. Assad J. A. (2006). Neurons in the orbitofrontal cortex encode economic value. Nature 441, 223–226. 10.1038/nature04676 16633341 PMC2630027

[B27] Parnaudeau S. O'Neil P. Bolkan S. S. Ward R. D. Abbas A. I. Roth B. L.. (2013). Inhibition of mediodorsal thalamus disrupts thalamofrontal connectivity and cognition. Neuron 77, 1151–1162. 23522049 10.1016/j.neuron.2013.01.038 PMC3629822

[B28] Parnaudeau S. Taylor K. Bolkan S. S. Ward R. D. Balsam P. D. Kellendonk C. (2015). Mediodorsal thalamus hypofunction impairs flexible goal-directed behavior. Biol. Psychiatry. 77, 445–453. 10.1016/j.biopsych.2014.03.020 24813335 PMC4177020

[B29] Paxinos G. Franklin K. G. J. (2001). The Mouse Brain in Stereotaxic Coordinates. San Diego, CA: Academic Press.

[B30] Pessoa L. Engelmann J. B. (2010). Embedding reward signals into perception and cognition. Front. Neurosci. 4:17 10.3389/fnins.2010.00017 20859524 PMC2940450

[B31] Pessoa L. Kastner S. Ungerleider L. G. (2003). Neuroimaging studies of attention: from modulation of sensory processing to top-down control. J. Neurosci. 23, 3990–3998. 12764083 10.1523/JNEUROSCI.23-10-03990.2003 PMC6741071

[B32] Robbins T. W. (2002). The 5-choice serial reaction time task: behavioural pharmacology and functional neurochemistry. Psychopharmacology 163, 362–380. 10.1007/s00213-002-1154-7 12373437

[B33] Rudebeck P. H. Murray E. A. (2014). The orbitofrontal oracle: cortical mechanisms for the prediction and evaluation of specific behavioral outcomes. Neuron 84, 1143–1156. 10.1016/j.neuron.2014.10.049 25521376 PMC4271193

[B34] Saddoris M. P. Gallagher M. Schoenbaum G. (2005). Rapid associative encoding in basolateral amygdala depends on connections with orbitofrontal cortex. Neuron 46, 321–331. 10.1016/j.neuron.2005.02.018 15848809

[B35] Salamone J. D. Correa M. Farrar A. Mingote S. M. (2007). Effort-related functions of nucleus accumbens dopamine and associated forebrain circuits. Psychopharmacology 191, 461–482. 10.1007/s00213-006-0668-9 17225164

[B36] Schoenbaum G. Chiba A. A. Gallagher M. (1998). Orbitofrontal cortex and basolateral amygdala encode expected outcomes during learning. Nat. Neurosci. 1, 155–159. 10.1038/407 10195132

[B37] Schoenbaum G. Chiba A. A. Gallagher M. (1999). Neural encoding in orbitofrontal cortex and basolateral amygdala during olfactory discrimination learning. J. Neurosci. 19, 1876–1884. 10024371 10.1523/JNEUROSCI.19-05-01876.1999 PMC6782178

[B38] Schoenbaum G. Roesch M. R. Stalnaker T. A. Takahashi Y. K. (2009). A new perspective on the role of the orbitofrontal cortex in adaptive behavior. Nat. Rev. Neurosci. 10, 885–892. 10.1038/nrn2753 19904278 PMC2835299

[B39] Schoenbaum G. Saddoris M. P. Stalnaker T. A. (2007). Reconciling the roles of orbitofrontal cortex in reversal learning and the encoding of outcome expectancies. Ann. N.Y. Acad. Sci. 1121, 320–335. 10.1196/annals.1401.001 17698988 PMC2430624

[B40] Schoenbaum G. Takahashi Y. Liu T. L. McDannald M. A. (2011). Does the orbitofrontal cortex signal value? Ann. N.Y. Acad. Sci. 1239, 87–99. 10.1111/j.1749-6632.2011.06210.x 22145878 PMC3530400

[B41] Small D. M. Gitelman D. Simmons K. Bloise S. M. Parrish T. Mesulam M. M. (2005). Monetary incentives enhance processing in brain regions mediating top-down control of attention. Cereb. Cortex 15, 1855–1865. 10.1093/cercor/bhi063 15746002

[B42] Stott J. J. Redish A. D. (2014). A functional difference in information processing between orbitofrontal cortex and ventral striatum during decision-making behavior. Philos. Trans. R. Soc. Lond. B Biol. Sci. 369:20130472 10.1098/rstb.2013.0472 25267815 PMC4186226

[B43] Takahashi Y. K. Chang C. Y. Lucantonio F. Haney R. Z. Berg B. A. Yau H.. (2013). Neural estimates of imagined outcomes in the orbitofrontal cortex drive behavior and learning. Neuron 80, 507–518. 10.1016/j.neuron.2013.08.008 24139047 PMC3806218

[B44] Tremblay L. Schultz W. (1999). Relative reward preference in primate orbitofrontal cortex. Nature 398, 704–708. 10.1038/19525 10227292

[B45] van der Meer M. A. Redish A. D. (2009). Covert expectation-of-reward in rat ventral striatum at decision points. Front. Integr. Neurosci. 3:1 10.3389/neuro.07.001.2009 19225578 PMC2644619

[B46] van Duuren E. Escamez F. A. Joosten R. N. Visser R. Mulder A. B. Pennartz C. M. (2007). Neural coding of reward magnitude in the orbitofrontal cortex of the rat during a five-odor olfactory discrimination task. Learn. Mem. 14, 446–456. 10.1101/lm.546207 17562896 PMC1896094

[B47] Walton M. E. Behrens T. E. J. Buckley M. J. Rudebeck P. H. Rushworth M. F. S. (2010). Separable learning systems in the macaque brain and the role of the orbitofrontal cortex in contingent learning. Neuron 65, 927–939. 10.1016/j.neuron.2010.02.027 20346766 PMC3566584

[B48] Waltz J. A. Gold J. M. (2007). Probabilistic reversal learning impairments in schizophrenia: further evidence of orbitofrontal dysfunction. Schizophr. Res. 93, 296–303. 10.1016/j.schres.2007.03.010 17482797 PMC2063592

[B49] Wang X. Xia M. Lai Y. Dai Z. Cao Q. Cheng Z.. (2014). Disrupted resting-state functional connectivity in minimally treated chronic schizophrenia. Schizophr. Res. 156, 150–156. 10.1016/j.schres.2014.03.033 24794395

[B49a] Ward R. D. Simpson E. H. Richards V. L. Deo G. Taylor K. Glendinning J. I.. (2012). Dissociation of hedonic reaction to reward and incentive motivation in an animal model of the negative symptoms of schizophrenia. Neuropsychopharmacology 37, 1699–1707. 10.1038/npp.2012.15 22414818 PMC3358738

[B50] Ward R. D. Winiger V. Higa K. K. Kahn J. B. Kandel E. R. Balsam P. D.. (2015). The impact of motivation on cognitive performance in an animal model of the negative and cognitive symptoms of schizophrenia. Behav. Neurosci. 129, 292–299. 10.1037/bne0000051 25914923 PMC4880016

